# Non-Variceal Upper Gastrointestinal Bleeding: A Retrospective Cohort of 364 Cases, Historical Comparison, and Updated Management Algorithm

**DOI:** 10.3390/life15081320

**Published:** 2025-08-20

**Authors:** Laurențiu Augustus Barbu, Liviu Vasile, Liliana Cercelaru, Valeriu Șurlin, Stelian-Stefaniță Mogoantă, Gabriel Florin Răzvan Mogoș, Tiberiu Stefăniță Țenea Cojan, Nicolae-Dragoș Mărgăritescu, Anca Buliman

**Affiliations:** 1 Department of Surgery, Railway Clinical Hospital Craiova, University of Medicine and Pharmacy of Craiova, 2 Petru Rares Street, 200349 Craiova, Romania; laurentiu.barbu@umfcv.ro (L.A.B.); gabriel.mogos@umfcv.ro (G.F.R.M.); tiberiu.tenea@umfcv.ro (T.S.Ț.C.); 2 Department of Surgery, Emergency County Hospital, University of Medicine and Pharmacy of Craiova, 2 Petru Rares Street, 200349 Craiova, Romania; vliviu777@yahoo.com (L.V.); vsurlin@gmail.com (V.Ș.); ssmogo@yahoo.com (S.-S.M.); 3 Department of Embryology and Anatomy, University of Medicine and Pharmacy of Craiova, 200349 Craiova, Romania; liliana.cercelaru@umfcv.ro; 4 Department of Radiology, Faculty of Medicine, Titu Maiorescu University, 67A Gheorghe Petrașcu Street, 031593 Bucharest, Romania; buliman_anca@yahoo.com

**Keywords:** non-variceal upper gastrointestinal bleeding, peptic ulcer, retrospective cohort, endoscopic therapy, surgical intervention, risk factors, historical comparison, management algorithm

## Abstract

**Background**: Non-variceal upper gastrointestinal bleeding (NVUGIB) remains a critical medical–surgical emergency associated with significant morbidity, mortality, and healthcare burden worldwide. Despite advances in diagnostic and therapeutic modalities, NVUGIB continues to pose complex clinical challenges, particularly in resource-limited settings. **Methods**: This retrospective observational study analyzed 364 consecutive adult patients diagnosed with NVUGIB and hospitalized at the First Surgical Clinic of the County Emergency Clinical Hospital Craiova between January 2009 and December 2014. Inclusion criteria required a confirmed diagnosis based on clinical presentation, laboratory findings, and upper gastrointestinal endoscopy (UGIE). Demographic variables, etiology, comorbidities, drug-induced triggers, laboratory parameters, onset-to-admission and onset-to-surgery intervals, endoscopic findings, therapeutic interventions (medical, endoscopic, surgical), rebleeding rates, and mortality were recorded and analyzed. Results were descriptively compared with historical data from the national and international literature. Due to the retrospective and aggregate nature of the data, survival analysis (Kaplan–Meier) was not applicable. **Results**: Peptic ulcers, erosive gastritis, Mallory–Weiss syndrome, and gastric neoplasms were the predominant etiologies. NSAID use, oral anticoagulation, and alcohol consumption emerged as major risk factors. Endoscopic hemostasis was achieved in the majority of cases; surgical intervention was required in 11.5% of patients, mainly for refractory or recurrent bleeding. The overall mortality rate was 10.9%, consistent with historical benchmarks. Comparative analysis revealed trends in etiology and management reflecting evolving clinical practice standards. **Conclusions**: NVUGIB remains a significant clinical challenge with persistent mortality and rebleeding risks. This cohort highlights the need for timely diagnosis, risk stratification, and an evidence-based therapeutic strategy integrating modern endoscopic and surgical options. An updated diagnostic and management algorithm is proposed to guide practical decision-making and optimize outcomes in similar tertiary care settings.

## 1. Introduction

Upper gastrointestinal bleeding (UGIB), defined as hemorrhage originating proximal to the ligament of Treitz, typically presents with hematemesis, coffee-ground emesis, or melena and remains a common medical emergency [1]. It accounts for over 300,000 hospital admissions annually in the United States, with mortality ranging from 3.5% to 10%, particularly affecting older adults and males [2]. While the incidence has declined due to a reduction in peptic ulcer disease (PUD), rebleeding remains a concern and is closely associated with increased mortality [3].

The most frequent causes of non-variceal UGIB are PUD (20–50%), gastroduodenal erosions (8–15%), esophagitis (5–15%), Mallory–Weiss tears (8–15%), vascular malformations, and neoplastic lesions [4,5]. Major risk factors include *Helicobacter pylori* infection, NSAID use, stress-related mucosal injury, and acid hypersecretion. Notably, *H. pylori*-positive ulcers are associated with better outcomes, while idiopathic ulcers (negative for NSAIDs and *H. pylori*) correlate with increased severity and systemic disease burden [5,6].

Endoscopic therapy remains the standard of care, with injection (e.g., epinephrine), thermal (e.g., argon plasma coagulation), and mechanical methods (e.g., hemoclips) applied based on lesion characteristics [7,8,9,10]. Novel agents such as TC-325 (Hemospray) provide non-contact hemostasis but are primarily used as adjuncts due to their transient mucosal effect [11,12,13,14,15].

Despite advances in diagnostic and therapeutic techniques, variability in outcomes persists across populations, and regional data from Eastern Europe—particularly Romania—remain limited and dispersed. National studies conducted more than a decade ago nonetheless provide a valuable basis for examining practice patterns and factors associated with outcomes in real-world settings.

The objective of this study was to analyze the clinical features, management strategies, and outcomes of patients with non-variceal upper gastrointestinal bleeding (NVUGIB) admitted to our center. A comparative assessment with data from the Romanian and international literature was conducted to identify relevant differences in epidemiology, treatment approaches, and prognostic factors. This analysis aimed to enhance the understanding of NVUGIB patterns in our setting and support evidence-based improvements in patient care.

## 2. Materials and Methods

### 2.1. Study Design and Setting

This retrospective observational study included 364 adult patients diagnosed with non-variceal upper gastrointestinal bleeding (NVUGIB), admitted to the First Surgical Clinic of the Emergency County Clinical Hospital of Craiova between 1 January 2009 and 31 December 2014.

### 2.2. Inclusion and Exclusion Criteria

Inclusion criteria comprised patients aged ≥18 years with a confirmed diagnosis of NVUGIB based on clinical presentation, laboratory data, and upper gastrointestinal endoscopy (UGIE). Exclusion criteria included variceal upper gastrointestinal bleeding, lower gastrointestinal bleeding, incomplete medical records, or comorbidities that could significantly confound bleeding etiology assessment.

### 2.3. Data Collection

The following variables were recorded for each patient:

- **demographics**: age, sex;
- **bleeding etiology** and **comorbidities**: cardiovascular, pulmonary, renal, and hepatic diseases;
- **risk factors**: prior peptic ulcer, gastritis, Mallory–Weiss syndrome, liver cirrhosis, chronic alcohol use, NSAIDs, acetylsalicylic acid, paracetamol, oral anticoagulants.

Pre-admission medications were abstracted verbatim from clinical charts and emergency department notes. Records did not consistently capture dose, duration, or concomitant use; therefore, each agent was categorized individually, and simultaneous NSAID co-use could not be confirmed.

- **Baseline laboratory parameters**: hemoglobin, hematocrit, platelet count, creatinine, urea;
- **timing variables**: onset-to-admission, admission-to-endoscopy, and onset-to-surgery intervals;
- **endoscopic findings**, lesion types and classification;
- **therapeutic interventions**: medical, endoscopic, and surgical;
- **clinical outcomes**: mortality, rebleeding, and drug-related bleeding.

### 2.4. Endoscopic and Surgical Management

All patients underwent UGIE within 24 h of admission, following ESGE guidelines. Therapeutic procedures included adrenaline injection and hemoclip placement. Surgery was reserved for patients with failed conservative management or recurrent bleeding.

Patients on oral anticoagulants (vitamin K antagonists or direct oral anticoagulants) were managed according to international guidelines. This included temporary cessation of anticoagulation, intravenous PPI therapy, and, when necessary, administration of prothrombin complex concentrate (PCC) or fresh frozen plasma (FFP), alongside tailored fluid and transfusion therapy.

### 2.5. Statistical Analysis

Descriptive statistics were reported as mean ± standard deviation (SD) for continuous variables. Due to the absence of time-to-event data, survival analyses were not performed. Comparative analyses with national and international data (e.g., Alharabi, Romcea, Matei, Botianu, Groza) were descriptively conducted. Inferential statistical tests included Student’s *t*-test and Chi-square tests. A *p*-value < 0.05 was considered statistically significant. Data were processed using Microsoft Excel 2019 (Microsoft Corp., Redmond, WA, USA) with XLSTAT (Addinsoft SARL, Paris, France).

### 2.6. Ethics Statement

This study was conducted in accordance with the Declaration of Helsinki and was approved by the Ethics Committee of the Emergency County Clinical Hospital of Craiova (approval number 32909/14 July 2025). Informed consent for the use of medical data in research was obtained from all patients at admission, as part of the standard hospital documentation. This included patients who later died. All data were anonymized prior to analysis.

## 3. Results

The results section presents a detailed comparative analysis of our study population and the findings reported in the relevant literature. The data include demographic characteristics, clinical and endoscopic findings, baseline laboratory parameters, triggering factors, therapeutic approaches (medical, endoscopic, and surgical), as well as outcome measures such as mortality and rebleeding rates.

**Figure 1:**
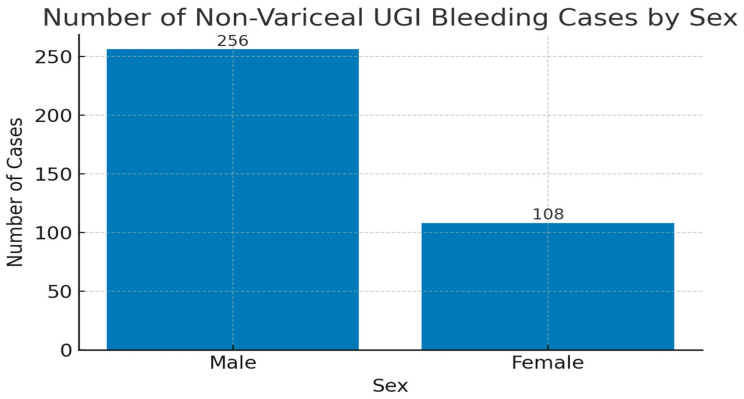
Sex distribution of patients with non-variceal upper gastrointestinal bleeding reported in our study.

In our cohort, 364 patients were diagnosed with non-variceal upper gastrointestinal bleeding (NVUGIB). Of these, 256 were male (approximately 70%) and 108 were female (approximately 30%—Figure 1).

**Figure 2:**
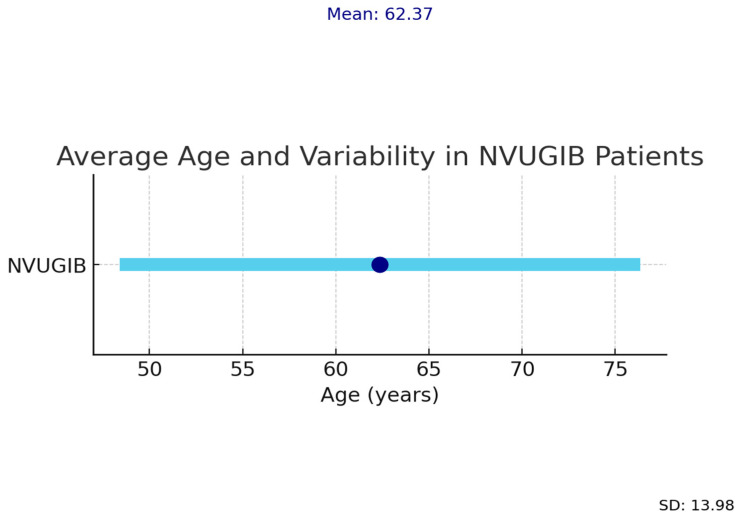
Mean age and standard deviation (SD) of patients with NVUGIB.

The mean age and standard deviation of patients with upper gastrointestinal bleeding in our study are shown in Figure 2.

**Figure 3:**
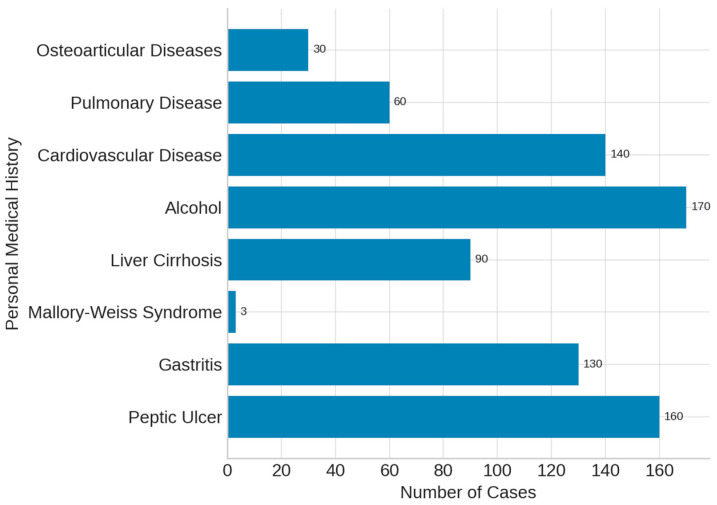
Patients’ personal medical history in UGIB.

Analysis of the patients’ personal medical history showed that the most frequent underlying condition was peptic ulcer disease, diagnosed in **192** patients (**52.7%**). Gastritis was reported in **132** cases, while liver cirrhosis was documented in **98** patients (**26.9%**). Alcohol consumption was recorded in **206** individuals (**56.6%**). Additional comorbidities included cardiovascular disease (**145** cases), pulmonary disease (**41** cases) and osteoarticular conditions (**28** cases). These findings are summarized in Figure 3.

**Figure 4:**
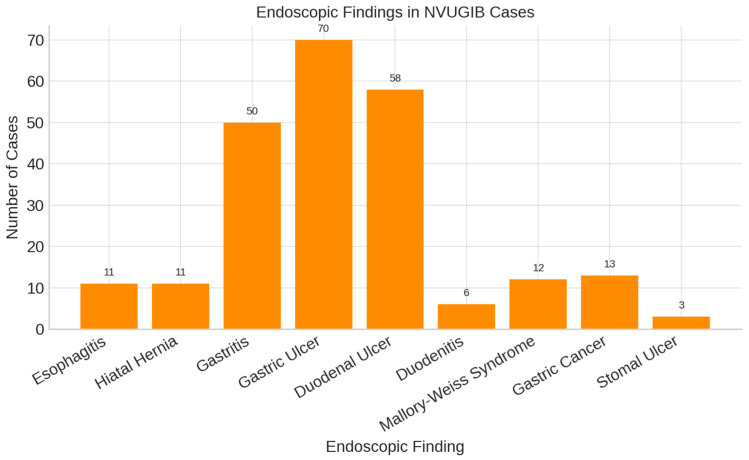
Endoscopic findings in NVUGIB.

Endoscopic evaluation in our cohort revealed that gastroduodenal ulcers were the predominant finding among patients with non-variceal upper gastrointestinal bleeding (Figure 4).

**Figure 5:**
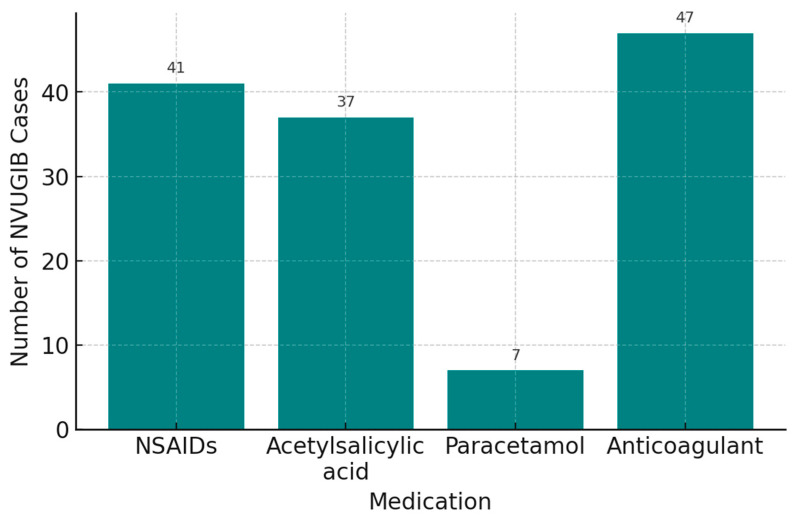
Medication use in NVUGIB cases. Legend: NSAIDs = non-steroidal anti-inflammatory drugs.

In our cohort, drug-related triggers for upper gastrointestinal bleeding included non-steroidal anti-inflammatory drugs (NSAIDs) in 36.2% of patients, acetylsalicylic acid in 7.1%, paracetamol in 1.9%, anticoagulants in 9.3%, and corticosteroids in 2.5%. Alcohol consumption was recorded in 56.6% of patients. These data are summarized in Figure 5. Information on concomitant NSAID use (e.g., acetylsalicylic acid with another NSAID) was unavailable.

**Figure 6:**
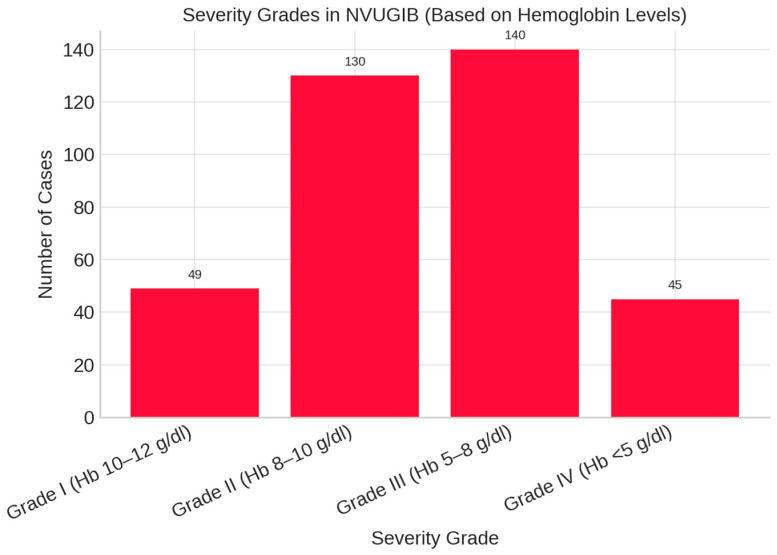
Severity grades in non-variceal upper gastrointestinal bleeding (based on hemoglobin levels).

Among the 364 patients with non-variceal upper gastrointestinal bleeding, 49 (13.5%) were classified as Grade I (Hb 10–12 g/dL), 130 (35.7%) as Grade II (Hb 8–10 g/dL), 140 (38.5%) as Grade III (Hb 5–8 g/dL), and 45 (12.4%) as Grade IV (Hb < 5 g/dL). Grade III was the most frequent severity category observed (Figure 6).

**Figure 7:**
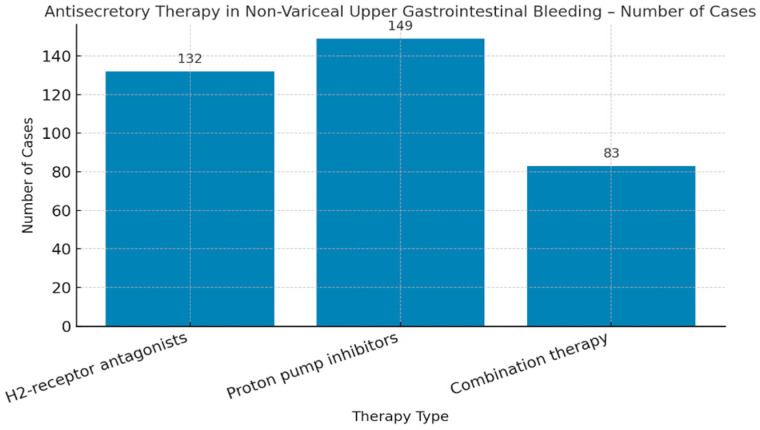
Distribution of antisecretory therapy types administered in non-variceal upper gastrointestinal bleeding. Legend: H2-receptor antagonists = histamine type 2 receptor antagonists.

The type of antisecretory therapy administered in non-variceal upper gastrointestinal bleeding was analyzed, with the distribution of patients receiving H2-receptor antagonists, proton pump inhibitors, or combination therapy (Figure 7).

**Figure 8:**
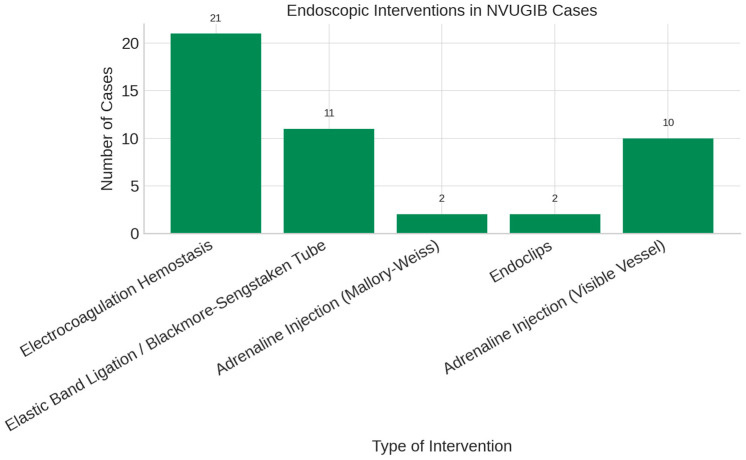
Endoscopic interventions performed in non-variceal upper gastrointestinal bleeding cases.

In our study, bipolar electrocoagulation was the most frequently used endoscopic therapy for non-variceal upper gastrointestinal bleeding (Figure 8).

**Figure 9:**
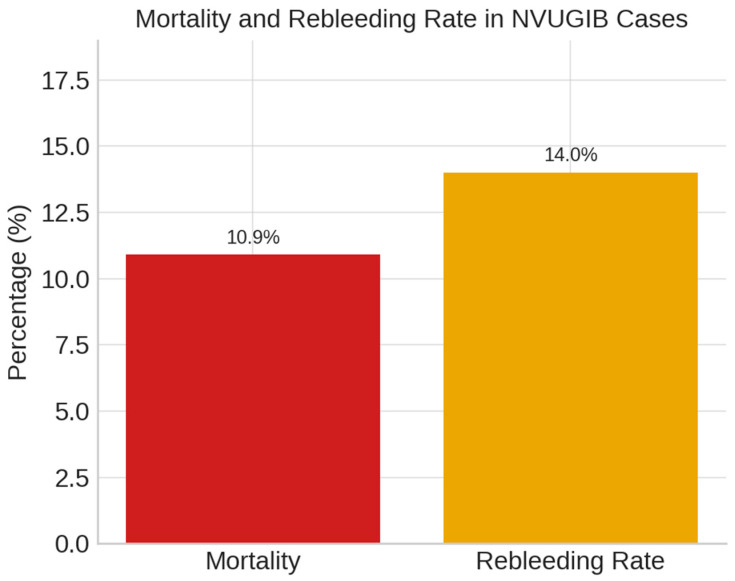
Mortality and rebleeding rate.

In our cohort, the mortality rate for non-variceal upper gastrointestinal bleeding was 10.9%, as shown in Figure 9.

**Figure 10:**
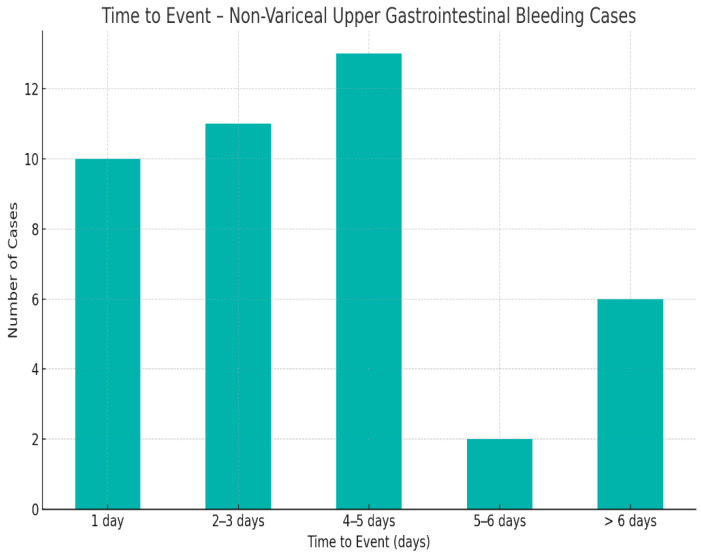
Distribution of cases by onset-to-surgery interval.

An important factor influencing patient outcomes in non-variceal upper gastrointestinal bleeding is the timing of surgical intervention (Figure 10).

**Figure 11:**
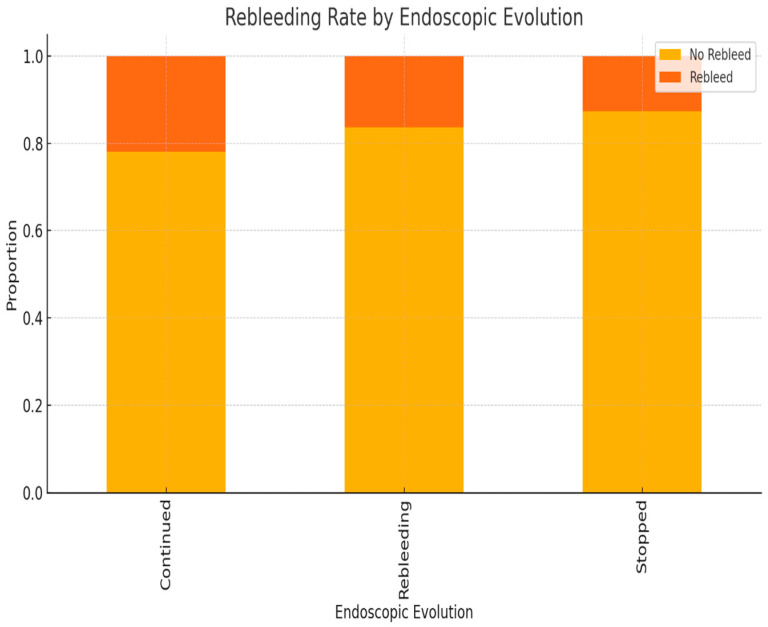
Proportion of patients with and without rebleeding, stratified by type of treatment (conservative vs. surgical).

Rebleeding occurred more frequently in patients who underwent surgical treatment; however, this difference was not statistically significant in univariate analysis (*p* > 0.05) (Figure 11).

**Figure 12:**
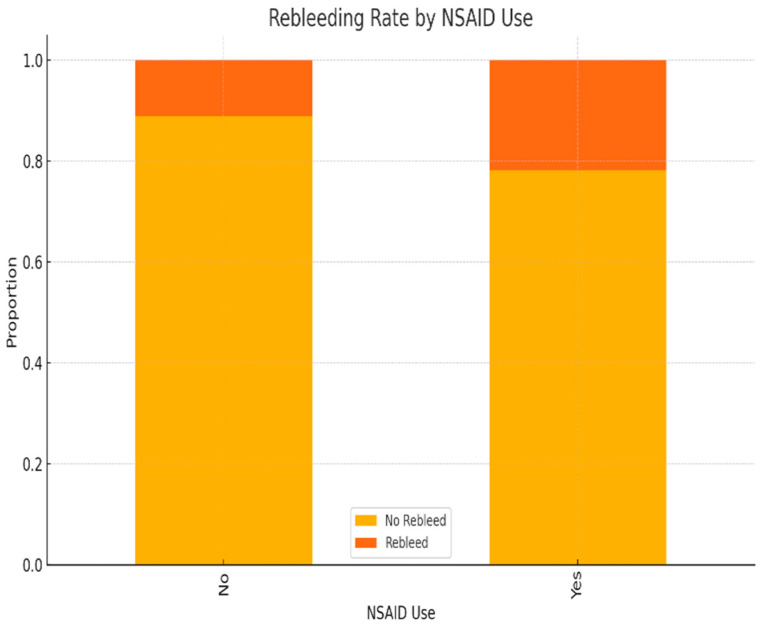
Comparison of rebleeding rates in patients with and without NSAID use.

NSAID use was associated with a higher proportion of rebleeding episodes. This difference reached statistical significance (χ^2^ test, *p* < 0.05), suggesting that NSAIDs may be an independent risk factor for rebleeding (Figure 12).

**Figure 13:**
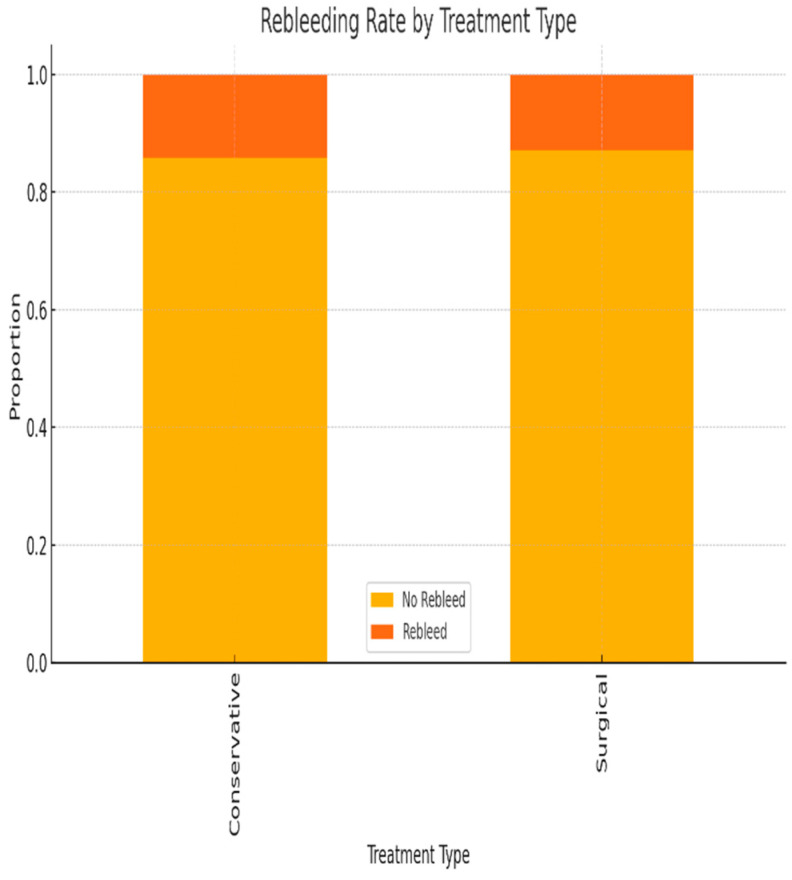
Rebleeding distribution based on the initial endoscopic evolution (bleeding stopped, continued, or recurred).

As expected, rebleeding was more frequently observed in patients whose bleeding had initially continued or recurred after endoscopic management. The association was statistically significant (*p* < 0.01) (Figure 13).

Multivariate logistic regression identified low hemoglobin at admission and alcohol use as independent predictors of rebleeding (*p* < 0.05). Endoscopic therapy was significantly associated with a reduced risk of rebleeding (*p* < 0.01). Age and NSAID use were not statistically significant in the multivariate model (Table 1).

**Figure 14:**
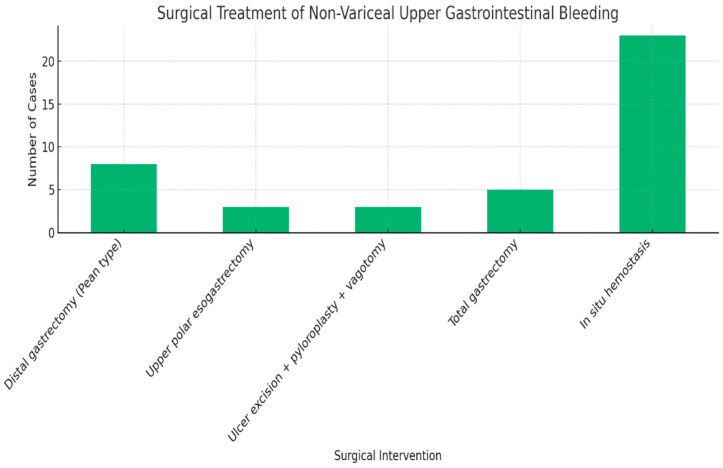
Surgical treatment of NVGIB.

The rate of surgical intervention for upper gastrointestinal bleeding was 11.5%. A comprehensive summary of the surgical techniques employed for non-variceal upper gastrointestinal bleeding, including the types of procedures and their respective case counts, is presented in Figure 14.

Multivariate analysis showed that rebleeding, low hemoglobin levels, and ulcerative lesions were significantly associated with the need for surgical intervention, while age and alcohol use were not (Table 2).

Multivariate analysis identified older age, low hemoglobin levels, alcohol use, anticoagulant therapy, and surgical intervention as independent predictors of in-hospital mortality (Table 3).

Student’s *t*-test and Chi-square tests were used for statistical comparisons. Patients who died were significantly older than survivors (68.4 ± 11.2 vs. 61.6 ± 14.1 years; *p* = 0.0005). Alcohol consumption was not significantly associated with mortality (*p* = 0.1001). Type of antisecretory therapy showed significant distribution differences (*p* = 0.0001), while trends in endoscopic therapy (*p* = 0.0911) and timing of surgery (*p* = 0.0565) did not reach statistical significance. Surgical procedure types differed significantly across categories (*p* < 0.0001) (Table 4).

## 4. Discussion

Upper gastrointestinal bleeding (UGIB) remains a critical medical–surgical emergency associated with significant morbidity, mortality, and healthcare burden, including in Eastern Europe [16,17,18]. Recent guidelines from the American College of Gastroenterology (ACG) have introduced key updates in the management of ulcer-related UGIB. These include a more selective approach to hospitalization, with outpatient management recommended for patients with a Glasgow-Blatchford Score (GBS) of 0–1, given their low risk of intervention or death. A restrictive transfusion strategy is also advised, with a hemoglobin threshold of 7 g/dL in stable patients, supported by evidence indicating lower rebleeding and mortality rates [18].

The ACG further recommends intravenous erythromycin prior to endoscopy to improve mucosal visualization and reduce the duration of hospitalization. Post-endoscopic care should involve high-dose proton pump inhibitor (PPI) therapy, either by continuous infusion or intermittent dosing, maintained for a minimum of 72 h. Additionally, advanced endoscopic techniques such as hemostatic powder spray (TC-325) and over-the-scope clips (OTSCs) are recognized as effective rescue tools for controlling active or recurrent bleeding [18].

In the present study, non-variceal UGIB was diagnosed in 364 patients, reflecting a high incidence consistent with the previous literature. Although data collection occurred before the widespread adoption of newer hemostatic methods and guideline updates, this study offers valuable real-world insight into clinical management within a tertiary care setting in Eastern Europe. Notably, male patients were nearly twice as frequently affected as females, likely due to increased exposure to modifiable risk factors including NSAID use, alcohol consumption, and smoking (Figure 1). This distribution aligns with prior European and global findings.

Incidence increased with age in our cohort, differing from findings by Pongprasobchai et al. [19], who observed stable rates across age groups, and Matei et al. [20], who reported overall lower incidence (Figure 2).

From patients’ medical history, the main causes of non-variceal UGIB were gastroduodenal ulcers, gastritis, Mallory–Weiss syndrome, and liver cirrhosis. Key risk factors included alcohol use and comorbidities such as cardiovascular, pulmonary, and osteoarticular diseases. These findings align with Alharbi et al. [16], who also identified ulcers and cirrhosis as leading etiologies (Figure 3).

The predominance of gastroduodenal ulcers in our cohort is consistent with both Romanian and international data, where duodenal ulcer is the leading cause of non-variceal UGIB (Figure 4). Erosive gastritis is also common, though its prevalence varies due to differences in diagnostic methods and populations. Rare lesions, such as Dieulafoy’s lesion and angiodysplasia, were infrequently observed. Overall, major etiologies appear consistent across studies, with variations shaped by local epidemiology and healthcare practices.

Although not statistically significant, the slightly higher rebleeding rate observed among surgically treated patients may reflect more advanced disease at presentation or suboptimal response to initial endoscopic management (Figure 11).

Our findings support the well-established role of NSAIDs as a risk factor for upper gastrointestinal mucosal damage and recurrence of bleeding, underlining the need for careful risk stratification and gastroprotective strategies in high-risk patients (Figure 12).

These findings reinforce the prognostic value of persistent or recurrent bleeding following endoscopic intervention and highlight the importance of close monitoring and timely escalation of care (Figure 13).

Our findings confirm that low hemoglobin levels and alcohol consumption significantly increase the risk of rebleeding, in line with previous reports. The protective effect of endoscopic therapy was also consistent with established data (Table 1).

The increased likelihood of surgical intervention in patients with rebleeding, low hemoglobin levels, or ulcerative lesions emphasizes the role of early risk stratification. Age and alcohol use did not reach statistical significance, suggesting a lesser impact on surgical decision-making in our cohort (Table 2).

These findings suggest that advanced age, anemia, alcohol use, and anticoagulant therapy contribute significantly to mortality risk in upper gastrointestinal bleeding. The association with surgical intervention may reflect the severity of cases requiring operative management. Early recognition of these high-risk factors is essential for optimizing patient outcomes (Table 3).

Older age was significantly associated with in-hospital mortality, confirming its role as a key risk factor in UGIB. Although alcohol use did not reach statistical significance, the observed trend suggests a potential contribution. The significant variation in antisecretory therapy and surgical procedures underscores the impact of individualized treatment strategies on clinical outcomes (Table 4).

### 4.1. Anticoagulation and Endoscopic Timing

Upper gastrointestinal bleeding (UGIB) in patients on chronic oral anticoagulants poses a therapeutic dilemma, requiring careful risk–benefit assessment between bleeding and thromboembolism. UGIB occurs in up to 20% of anticoagulated patients, with endoscopy essential for diagnosis and management, despite the absence of a consistent bleeding site.

Direct oral anticoagulants (DOACs), widely used for atrial fibrillation and venous thromboembolism, carry a bleeding risk comparable to or higher than warfarin. Although DOACs offer predictable pharmacokinetics and rapid clearance, they lack routine monitoring tools and universally available reversal agents. In acute UGIB, therapy should be promptly discontinued, with time serving as a key mitigating factor due to short half-lives. PCCs or activated PCCs may be used in severe cases; hemodialysis is effective only for dabigatran. Current guidance is largely expert-based, highlighting the need for robust clinical data [21,22,23,24].

In major bleeding, the American College of Clinical Pharmacology recommends prompt anticoagulant cessation and reversal with prothrombin complex concentrate (PCC) over fresh frozen plasma (FFP), along with 5–10 mg IV vitamin K [25]. PCCs, containing factors II, VII, IX, and X, offer rapid correction and are available in inactive or activated forms.

Conversely, the American College of Cardiology advises using FFP—without vitamin K—in patients with mechanical valves due to thrombosis risk [26].

Gastrointestinal bleeding under vitamin K antagonist (VKA) therapy requires urgent reversal, especially in unstable patients or those with high INR. ESGE guidelines favor PCC over FFP due to faster INR normalization, lower volume, and better bleeding control during endoscopy [26,27,28].

In anticoagulated patients, vitamin K antagonists (VKAs) should be withheld and reversed under specialist supervision. Recent guidelines recommend that direct oral anticoagulants (DOACs) should also be temporarily discontinued in the setting of acute non-variceal upper gastrointestinal bleeding (NVUGIB); however, endoscopy should not be delayed due to ongoing anticoagulation [1]. In cases of severe or life-threatening bleeding, reversal agents such as idarucizumab (for dabigatran) or andexanet alfa (for factor Xa inhibitors), or prothrombin complex concentrate (PCC), may be considered—although the evidence supporting their use is limited [2].

#### Resumption Strategies After Hemostasis

The 2022 International Consensus recommends resuming DOACs within 48–72 h after successful endoscopic hemostasis, in line with observational data showing no increased rebleeding risk. Acetylsalicylic acid for secondary prevention should be resumed within 3 days, provided bleeding is controlled [29].

Our data confirm NSAIDs and alcohol as leading triggers of NVUGIB, consistent with other Romanian and international studies. Compared to Alharbi et al. [16], who reported higher rates for paracetamol and anticoagulants, our lower values may reflect local prescribing patterns. Minor differences in acetylsalicylic acid and corticosteroid use across studies likely result from population risk profiles and access to gastroprotective strategies (Figure 5). These laboratory findings are comparable to those reported by Matei [20], indicating similar clinical profiles among patients with NVUGIB.

The ESGE guidelines recommend early assessment of hemodynamic status and prompt resuscitation with crystalloids in unstable patients. A restrictive transfusion strategy is advised, targeting hemoglobin 7–9 g/dL, with higher thresholds for those with cardiac comorbidities. Risk stratification, especially via the Glasgow-Blatchford Score (GBS), is essential to guide early discharge or urgent intervention [28].

### 4.2. Pharmacological Therapy

Proton pump inhibitors (PPIs) were the primary antisecretory therapy, used in 41% of non-variceal UGIB cases (Figure 7), while 36% received H2-receptor antagonists (H2RAs), and 23% a combination of both. These data reflect the preference for PPIs as first-line treatment.

H2RAs, though historically used, show limited efficacy in acute peptic ulcer bleeding due to rapid tolerance development and reduced acid suppression over time [30]. Comparative studies have shown PPIs maintain gastric pH more effectively than H2RAs. In our cohort, outcomes were similar between treatment groups, limiting definitive conclusions on superiority.

High-dose intravenous PPI therapy (80 mg bolus followed by 8 mg/h infusion for 72 h) has proven effective post-endoscopic treatment in patients with high-risk bleeding stigmata. Lau et al. reported a significant rebleeding reduction—from 22.5% to 6.7%—with PPI therapy [31].

In contrast, a large study (n = 1256) found no significant outcome difference between high-dose pantoprazole and ranitidine, though pantoprazole showed better overall efficacy. The unusually low rebleeding rate in the ranitidine group (3.2%) may explain this discrepancy [32].

A meta-analysis by Leontiadis et al. (24 RCTs, n = 4373) confirmed that PPIs significantly reduce rebleeding, surgery, repeat endoscopy, and mortality in non-variceal UGIB [33]. Similar benefits were reported by Laine et al., particularly with high-dose IV PPI therapy versus placebo [10].

Current guidelines support an 80 mg IV bolus followed by 8 mg/h infusion for 72 h, even without prior endoscopy, especially in patients with adherent clots [10,34]. Although Andriulli et al. found no dose-dependent differences, their results are limited by study design [34]. A meta-analysis by Wang et al. (7 RCTs, n = 1157) also found no clear advantage of high-dose over standard-dose PPI post-endoscopy [35].

Despite mixed findings, international consensus continues to recommend high-dose PPI infusion post-endoscopic hemostasis, which aligns with our clinical practice [31].

Pre-endoscopic PPI therapy reduces high-risk stigmata and the need for intervention but has no clear effect on rebleeding, surgery, or mortality. Tranexamic acid may reduce mortality, though evidence is outdated and inconsistent. Somatostatin and octreotide offer limited benefit and are not routinely recommended. IV erythromycin improves gastric visualization and reduces transfusions, repeat endoscopy, and hospital stay. Metoclopramide is less studied and poses neurological risks, warranting cautious use [36,37].

In the 1980s, investigators such as Graham [38] and Peterson [39] questioned the routine use of endoscopy in upper gastrointestinal bleeding (UGIB) due to the limited availability of effective therapeutic interventions at the time. This perspective shifted following a landmark 1990 meta-analysis by Sacks et al. [40], which demonstrated that endoscopic hemostasis significantly reduced rebleeding, the need for surgery, and mortality. This established endoscopy as a cornerstone in UGIB management. However, concerns about the safety of early endoscopy emerged in the mid-1990s, as studies by Yen [41] and Hill [42] reported higher rates of oxygen desaturation during urgent procedures in high-risk, unsedated patients without oxygen supplementation—conditions that differ substantially from current clinical practice.

Beyond safety, routine early endoscopy poses logistical and financial challenges. Maintaining 24/7 endoscopy services requires substantial staffing and infrastructure, especially in non-tertiary centers. Additionally, early procedures may detect transient stigmata, leading to potentially unnecessary interventions and increased costs.

Despite initial concerns, subsequent evidence supports the benefit of early endoscopy (within 24 h) in most patients with UGIB. Meta-analyses and cohort studies show that early intervention is associated with reduced hospital stay, lower transfusion needs, and fewer complications, particularly in high-risk patients [43,44,45,46].

According to the updated ESGE 2024 guideline, very early endoscopy (<6 h) is recommended only in unstable patients, while routine early endoscopy (<24 h) remains the standard of care. Additionally, topical hemostatic agents such as Hemospray are now recognized as temporary rescue options when conventional endoscopic methods fail [30].

Massive UGIB requires emergency endoscopy after hemodynamic stabilization; in active bleeding, it may proceed during resuscitation. Caution is warranted in elderly and comorbid patients due to reduced tolerance to hypovolemia and increased risk of organ dysfunction.

Standard management includes endoscopic hemostasis followed by acid suppression, which lowers rebleeding risk and improves outcomes [43]. Gastric acidity impairs clot stability by inhibiting platelet function and promoting fibrinolysis. A gastric pH > 4–6 supports hemostasis, while pH < 2 activates pepsin, leading to clot lysis [44,45].

The rationale for acid suppression in UGIB lies in the negative impact of gastric acid on clot stability, including:

- inhibition of platelet aggregation;
- clot lysis via pepsin activation at low pH;
- enhanced fibrinolysis in acidic environments;
- optimal clot stability at pH 6–6.5, as shown in experimental models [46].

### 4.3. Endoscopic Therapy

Endoscopic therapy is the first-line treatment for bleeding gastroduodenal ulcers, including massive hemorrhage. It enables source identification, excludes variceal bleeding, and guides risk stratification via endoscopic stigmata. Early endoscopy improves outcomes, especially in cases with active bleeding or visible vessels.

ESGE recommends endoscopy within 24 h, or <12 h for high-risk patients. The Forrest classification is used to guide therapy: Forrest Ia, Ib, IIa, and IIb (after clot removal) require hemostasis, while IIc and III do not. Epinephrine should be combined with mechanical or thermal methods, not used alone [30].

For non-ulcer lesions (e.g., Mallory–Weiss, Dieulafoy, angioectasias, tumors), endoscopic therapy is recommended despite limited long-term data. If endoscopy fails or is not feasible, transcatheter angiographic embolization (TAE) or surgery should be considered [47].

Risk stratification in acute UGIH is critical for guiding intervention and determining inpatient vs. outpatient care. The Glasgow-Blatchford Score (GBS) and Rockall score are widely validated, predicting the need for intervention and mortality, respectively.

Recent APAGE recommendations support incorporating serum lactate levels alongside clinical scoring systems such as the Glasgow-Blatchford Score to better stratify high-risk patients with UGIB [48].

In our cohort, interventional endoscopy was performed in 19.8% of cases—lower than rates reported by Botianu et al. [49] (Figure 9). Techniques included epinephrine injection, hemoclips, and bipolar electrocoagulation in select cases, underscoring its role when pharmacologic therapy is insufficient.

A meta-analysis by Cook et al. (30 trials) confirmed that endoscopic therapy significantly reduces rebleeding, surgery, and mortality, particularly in patients with active bleeding or visible vessels [50]. Kahi et al. later showed that endoscopic treatment of ulcers with adherent clots reduced rebleeding (8.2%) compared to medical therapy alone (24.7%) [51].

Adding a second endoscopic modality during the same session reduces rebleeding (18.8% to 10.4%), need for surgery (10.8% to 7.1%), and mortality (5% to 2.5%), regardless of technique. Hemoclips and thermal coagulation are both effective, with hemoclips mimicking surgical ligation.

However, hemoclips may be technically limited in fibrotic ulcers, difficult angles, or multiple bleeding sites. In one RCT, deployment failure occurred in 10% of cases due to device-related issues [52].

### 4.4. Limitations of Endoscopic Therapy in Severe Gastrointestinal Bleeding

A key limitation of endoscopic hemostasis is bleeding from erosion into large-caliber arteries, where control is difficult. As per Poiseuille’s law, small increases in vessel diameter lead to exponential rises in flow, complicating endoscopic efficacy.

In a pre-endoscopy era study, Swain et al. analyzed 27 emergency gastrectomies for bleeding ulcers and found average arterial diameters of 0.7 mm (range: 0.1–1.8 mm). Nearly half were subserosal vessels, with some showing aneurysmal dilation, especially in large, penetrating ulcers. These anatomical factors present major challenges for endoscopic treatment [53].

In a follow-up study, Swain reported a mean arterial diameter of 3.75 mm at bleeding sites in fatal gastric ulcer cases, highlighting the limitations of endoscopic therapy in large-vessel erosion [53].

For high-risk NVUGIB (Forrest Ia, Ib, IIa), endoscopic hemostasis—via injection, thermal, or mechanical methods—is superior to pharmacotherapy alone. Meta-analyses support combination therapy (e.g., epinephrine plus clips or thermal coagulation) as the most effective strategy to reduce rebleeding and surgical need [30,54].

Topical agents such as Hemospray are now endorsed in the ESGE 2024 guideline as temporary rescue options when standard endoscopic modalities fail [55,56].

While erosive esophagitis, gastritis, and duodenitis often respond to high-dose PPI therapy without endoscopy, early endoscopy remains critical for risk stratification and treatment of Mallory–Weiss tears, Dieulafoy lesions, angioectasias, and tumors. In these cases, combined endoscopic modalities offer improved hemostasis, though mortality benefits remain uncertain and require further evidence.

### 4.5. Post-Endoscopic Management

In post-endoscopic management of non-variceal upper gastrointestinal hemorrhage (NVUGIH), ESGE recommends high-dose PPI therapy (intravenous bolus followed by 72-h infusion) after successful hemostasis or in cases with adherent clots. Intermittent IV boluses or high-dose oral PPIs may be used in patients able to take oral medication.

The ACG guidelines emphasize that high-dose intravenous PPIs should be reserved for patients with high-risk endoscopic stigmata, while patients with clean-based ulcers may be managed with oral PPI therapy and do not require second-look endoscopy [18].

In cases of clinical rebleeding, repeat endoscopy is advised, with angiographic embolization or surgery if re-hemostasis fails.

Testing for *Helicobacter pylori* should be performed during the acute phase, with eradication therapy if positive and later confirmation of clearance.

Anticoagulation should be resumed based on individual thromboembolic risk, typically within 7–15 days, or earlier in high-risk patients. Recent international guidelines (e.g., ESGE, BSG, and ACG) recommend that acetylsalicylic acid used for secondary cardiovascular prevention should not be discontinued during episodes of non-variceal upper gastrointestinal bleeding (NVUGIB). If temporarily interrupted, acetylsalicylic acid therapy should be resumed as soon as hemostasis is confirmed endoscopically—ideally within 3 to 5 days. In contrast, in cases where acetylsalicylic acid is prescribed for primary prevention, discontinuation may be considered, and resumption should be carefully re-evaluated based on individual risk–benefit balance. For patients on dual antiplatelet therapy (DAPT), acetylsalicylic acid should be maintained and the P2Y12 inhibitor restarted after endoscopic hemostasis in consultation with cardiology. PPI co-therapy is strongly recommended to minimize gastrointestinal risk [18,57,58].

Evidence-based guidelines for NVUGIH recommend high-dose PPI therapy (80 mg IV bolus followed by 8 mg/h infusion for 72 h) after endoscopic hemostasis, based on data showing reduced rebleeding, surgery, and mortality. However, recent meta-analyses, including that by Sachar et al., suggest that intermittent PPI dosing—oral or IV—may be non-inferior, though slightly higher rebleeding rates may require retreatment [32].

High-dose oral PPI regimens appear to achieve similar intragastric pH levels, but larger trials are needed to confirm their efficacy across populations with varying metabolism profiles [30,58].

Hemostatic powders and over-the-scope clips (OTSCs) are promising salvage options for refractory bleeding. Routine second-look endoscopy remains controversial due to limited benefit and cost-effectiveness, except in high-risk patients.

Peptic ulcer disease, primarily linked to *Helicobacter pylori*, remains the most frequent NVUGIH cause. Delayed testing improves accuracy, and eradication confirmation is essential to prevent recurrence.

Observational data support resuming anticoagulation around 7 days post-bleed to reduce thromboembolic risk and improve survival. Individualized decisions, including bridging strategies, may be necessary in high-risk patients [30,59].

In NVUGIH, acetylsalicylic acid can be safely resumed by day 3 in patients with high-risk endoscopic stigmata, balancing rebleeding risk with thrombotic prevention. In patients without high-risk stigmata, immediate resumption is appropriate, as PPIs support ulcer healing irrespective of antiplatelet use.

Although initial concerns existed about a potential interaction between clopidogrel and PPIs, recent randomized trials and propensity-matched studies show no significant increase in cardiovascular events. Meanwhile, PPI co-therapy effectively reduces GI bleeding, supporting its continued use in patients on dual antiplatelet therapy [30,47].

### 4.6. Predictive Factors for Endoscopic Therapy Failure in Upper Gastrointestinal Bleeding

Elmunzer et al. reviewed ten prospective studies on predictors of endoscopic therapy failure in upper gastrointestinal bleeding, identifying hemodynamic instability and significant comorbidities as key pre-endoscopic risk factors [60]. Intra-procedural predictors included active bleeding, large ulcer size, and ulcer location on the lesser curvature of the stomach or posterior wall of the duodenal bulb.

These locations are prone to erosion into major arteries such as the gastroduodenal or left gastric artery, often resulting in bleeding that exceeds the hemostatic capacity of current endoscopic methods. Therefore, hemodynamic instability, ongoing hemorrhage, large ulcer size, and posterior duodenal location are strong predictors of rebleeding and treatment failure, underscoring the anatomical and technical limitations of endoscopic intervention.

Non-variceal upper gastrointestinal bleeding should be assessed within a broader vascular context, as thrombotic vascular conditions such as ischemic colitis secondary to a thrombosed aortic aneurysm may indicate an underlying prothrombotic state with implications for both prognosis and therapeutic strategy [61].

### 4.7. The Role and Timing of Surgical Intervention in Upper Gastrointestinal Bleeding

Surgical intervention remains the definitive treatment for patients in whom endoscopic hemostasis fails or is not feasible. While often considered a clinical endpoint in studies on endoscopic efficacy, the need for surgery has markedly decreased with advances in endoscopic techniques and is now typically reserved for cases of life-threatening hemorrhage.

In our cohort, endoscopy was used for both diagnosis and treatment in 50.2% of gastroduodenal ulcer cases. Combined medical and endoscopic therapy was successful in 89.7% of patients, while 10.3% required surgery. Predictors of endoscopic failure included gastric ulcer location, large ulcer size, and hemodynamic instability—present in 41.6% of surgically managed cases.

Mortality after failed endoscopic therapy ranges between 15% and 25% [62]. Risk factors for failure include massive hemorrhage with hypotension, recurrent bleeding, advanced age, and unfavorable ulcer characteristics such as large size and difficult location. The optimal timing of surgical intervention remains debated, particularly since the 1980s.

Reported mortality rates for non-variceal upper gastrointestinal bleeding (NVUGIB) vary widely, from 2.6% [63] to 14% [64], depending on factors such as timeliness of endoscopy, use of standardized protocols, patient comorbidity, and access to specialized care.

In our study, the mortality rate was 10.9%, consistent with international reports ranging from 2.6% to 14% and reflecting comparable outcomes across diverse clinical settings. This closely aligns with the 10.43% mortality rate reported by Botianu [49], suggesting similar patient management strategies and risk profiles in Romania. Conversely, the higher mortality rate of 18.7% reported by Dicu [65] may be attributed to delayed interventions or differences in patient comorbidities.

Multivariate analysis revealed that increased age, low hemoglobin levels at admission, alcohol use, anticoagulant therapy, and surgical intervention were independently associated with in-hospital mortality. These findings emphasize the need for early recognition and aggressive management of high-risk patients.

Statistical analysis using *t*-tests and Chi-square tests confirmed that mortality was significantly associated with older age and type of antisecretory therapy. Timing of surgery approached statistical significance, while alcohol use did not show a statistically significant association with mortality. Tailored surgical approaches were significantly associated with patient outcome (Table 4).

Rebleeding rates showed notable variation, with our study (14%) closely aligning with the 12.02% reported by Botianu et al [49]. In contrast, Dicu [65] observed a significantly higher rate of 40.2%, likely reflecting differences in endoscopic technique, hemostasis protocols, and post-procedural care.

These findings underscore the persistent burden of mortality and rebleeding in non-variceal upper gastrointestinal bleeding and reinforce the need for improved diagnostic accuracy and standardized therapeutic strategies.

The Birmingham study evaluated 104 patients with bleeding peptic ulcers, comparing early versus delayed surgical intervention [66]. Early surgery was indicated by transfusion of ≥4 units in 24 h, rebleeding, high-risk endoscopic stigmata, or recent upper GI bleeding history. Delayed surgery was reserved for more extensive transfusion needs or repeated rebleeding.

In patients under 60, early surgery was performed in 52% compared to 5% in the delayed group. For those over 60, early surgery occurred in 62% versus 27%, indicating that both age and timing significantly influence the need for surgical intervention.

Applying similar criteria in our study, immediate surgery within 24 h was performed in 29.1% of cases, delayed emergency surgery (within 72 h) in 25%, and late interventions (beyond 72 h) in 45.9%. Among patients under 60 years, early surgery was required in 35% of cases, compared to 25% among those undergoing late intervention.

Saperas et al. evaluated 69 patients over 50 years old with non-arterial bleeding or stigmata of recent hemorrhage without a visible vessel, showing that neither early nor delayed surgery was required, supporting conservative management in the absence of high-risk endoscopic features [67].

These findings, along with prior data, suggest that early surgery should generally be avoided unless high-risk endoscopic stigmata are present. Endoscopic therapy remains the first-line treatment for peptic ulcer bleeding, with surgery reserved for refractory cases.

In rebleeding scenarios, the decision between repeat endoscopy and surgery remains debated. While some elderly, high-risk patients may benefit from a second-look endoscopy, persistent hypotension or multiple failed endoscopic attempts may delay definitive treatment. In such cases, timely surgical intervention may improve outcomes [66].

Cheung et al. treated 1169 patients with peptic ulcer bleeding using epinephrine injection followed by thermal coagulation over a 40-month period [66]. Endoscopic hemostasis failed in 17 patients who required surgery. The rebleeding rate after successful endoscopic therapy was 8.7%. No significant differences were observed in 30-day mortality, hospital stay, or transfusion requirements. However, surgical patients had a higher rate of post-operative complications. Repeat endoscopy controlled rebleeding in 75% of cases, indicating it may be a viable alternative to surgery in selected patients.

Ulcer size > 2 cm and hypotension at rebleeding were independent predictors of endoscopic failure. In our cohort, these factors were present in 11 cases (9.73% of ulcer patients), accounting for 32.3% of surgeries for peptic ulcer bleeding.

Imhof et al. compared endoscopic fibrin glue injection to early surgery in patients with arterial bleeding or visible vessels ≥ 2 mm in peptic ulcers [68]. Rebleeding occurred in 50% of the endoscopy group versus 4% in the surgery group, with no significant difference in mortality. Early surgery offered more effective and definitive hemostasis in high-risk cases.

Bender et al. advocated for surgery in patients presenting with hemorrhagic shock, especially those over 65 years, with ulcers > 2 cm, high-risk stigmata, or prior ulcer complications [69]. Among 66 surgically treated patients followed over five years, no mortality was reported.

In our cohort, 211 patients were over 65 years; 38 had hemorrhagic shock and underwent surgery for bleeding peptic ulcers, including 4 with ulcers > 2 cm. Only one post-operative complication (anastomotic fistula) occurred, managed conservatively.

Mueller et al. operated on 157 patients with spurting hemorrhage, visible vessels in posterior duodenal ulcers, or transfusion needs >6 units in 24 h, or rebleeding within 48 h. The reported 30-day post-operative mortality was 7% [70].

### 4.8. Type of Surgical Intervention in Peptic Ulcer Bleeding

In the current era of proton pump inhibitors and *Helicobacter pylori* eradication, surgery in peptic ulcer bleeding aims primarily at achieving hemostasis rather than a definitive cure.

Poxon et al. [71] studied 137 patients treated surgically for bleeding ulcers. Rebleeding occurred in 6 of 62 patients undergoing oversewing, but none following conventional procedures (e.g., ulcer excision with vagotomy or gastrectomy). The trial was halted due to high fatal rebleeding rates in conservative surgery groups, with mortality reaching 26% for oversewing and 19% for conventional surgery.

In our cohort, no rebleeding was observed after in situ hemostasis. Post-operative complications were mostly infectious, such as wound infections and fistulas.

Most surgeries occurred within 1–5 days of symptom onset, supporting a stepwise approach favoring initial medical and endoscopic therapy. Delayed surgeries (beyond six days) likely reflect initially successful conservative management or late-onset complications requiring surgical resolution (Figure 10).

In our cohort, 11.5% of patients with non-variceal upper gastrointestinal bleeding required surgery, slightly exceeding the 7.66% reported by Botianu et al. [49]. This difference may reflect variations in case severity, patient selection, or differences in the availability and effectiveness of endoscopic hemostasis prior to surgical intervention. Given the retrospective nature of our study and the study period (2009–2014), these findings also likely reflect the healthcare context in Eastern Europe at that time, when financial and infrastructural constraints limited uniform 24/7 access to functional endoscopic services, potentially leading to a higher proportion of surgical cases.

Among the 42 surgically treated patients in our series, in situ hemostasis was the most common approach (54.8%), reflecting a preference for limited, targeted bleeding control (Figure 14). Distal gastrectomy (19%) and ulcer excision with pyloroplasty and vagotomy (7%) were selectively performed, while extensive procedures such as total gastrectomy or upper polar esogastrectomy were reserved for complex cases. These data support a tailored surgical strategy focused on hemostasis and organ preservation.

Angiographic therapy was introduced in the 1970s as a surgical alternative for massive peptic ulcer bleeding [71], predating endoscopic hemostasis. Technological advances—such as microcatheters and improved embolic agents—have enhanced safety and durability, reducing ischemic complications.

Refractory ulcers often erode into major arteries: gastric ulcers along the lesser curvature may affect branches of the left gastric artery, while posterior duodenal ulcers typically involve the gastroduodenal or pancreaticoduodenal arteries [72].

Eriksson et al. introduced a technique using endoscopic clip placement to guide angiographic localization of bleeding sites [73]. Various embolic agents—such as cyanoacrylate, microspheres, and gelatin particles—can be used alone or in combination for vessel occlusion. Complications include gastric or duodenal infarction, hepatic ischemia, and delayed duodenal strictures [74], with adhesive agents requiring caution due to potential reflux into non-target vessels.

Angiographic embolization is effective in high-risk surgical candidates, after failed endoscopy, or in post-operative rebleeding. Technical success ranges from 90 to 100%, with clinical success between 50 and 83%.

Ripoll et al. compared surgery versus embolization, noting similar rebleeding (29% vs. 23%) and mortality rates (26% vs. 21%) despite older age and more comorbidities in the embolization group [75]. Eriksson et al. also reported lower 30-day mortality in embolized patients compared to those undergoing surgery after failed endoscopy, supporting embolization as a safer option in selected high-risk cases [73].

**Figure 15:**
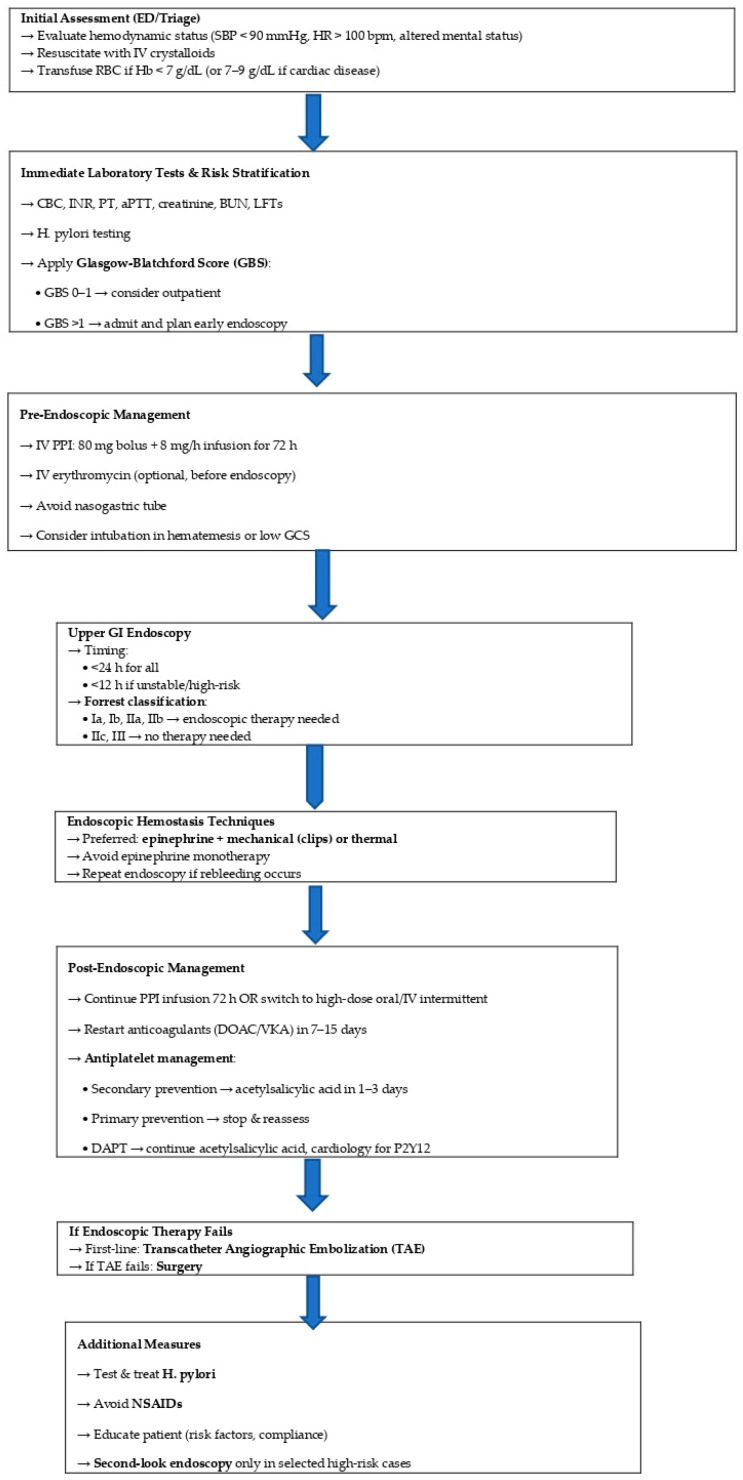
Stepwise approach to acute upper gastrointestinal bleeding.

Effective management of upper gastrointestinal bleeding (UGIB) requires timely, structured clinical decisions to reduce morbidity and mortality. We propose a practical stepwise algorithm to guide clinicians from emergency evaluation to post-endoscopic care (Figure 15). This approach integrates current evidence and best practices, emphasizing early risk stratification, optimal resource use, and individualized therapy.

### 4.9. Limitations

This study has several limitations. First, its retrospective design depends on the completeness and accuracy of medical records. Data on concurrent NSAID therapy—such as acetylsalicylic acid in combination with other non-steroidal anti-inflammatory drugs—were not systematically recorded, including dose and duration. Although comorbidities were documented, their influence on bleeding risk and patient outcomes was not explored in depth. These factors may affect the results and should be addressed in prospective studies with standardized data collection.

## 5. Conclusions

This retrospective study highlights that non-variceal upper gastrointestinal bleeding (NVUGIB) remains a significant clinical challenge, with peptic ulcer as the main cause. Independent risk factors for rebleeding and mortality were identified, including low hemoglobin at admission, alcohol use, advanced age, anticoagulant therapy, and the need for surgery. Endoscopic hemostasis significantly reduced the risk of rebleeding. Based on these findings, we propose an updated algorithm to optimize NVUGIB management, emphasizing early risk stratification, timely endoscopy, and individualized decisions regarding antithrombotic therapy.

## Figures and Tables

**Table 1:** Multivariate logistic regression analysis of factors associated with rebleeding in NVUGIB (n = 364).

Variable	Odds Ratio (OR)	95% Confidence Interval	*p*-Value
**Age (years)**	1.04	1.01–1.07	0.011
**Hemoglobin (g/dL)**	0.82	0.69–0.97	0.017
**Alcohol use (Yes vs. No)**	2.15	1.18–3.91	0.013
**Anticoagulant use (Yes vs. No)**	2.45	1.21–4.98	0.012
**Surgical intervention (Yes)**	2.92	1.32–6.48	0.008

Legend: *p*-value = probability value.

**Table 2:** Multivariate logistic regression analysis of factors associated with surgical intervention in NVUGIB (n = 364).

Variable	Odds Ratio (OR)	95% Confidence Interval	*p*-Value
**Age (years)**	1.01	0.97–1.05	0.510
**Hemoglobin (g/dL)**	0.75	0.61–0.93	0.008
**Rebleeding (Yes vs. No)**	3.90	2.00–7.60	<0.001
**Ulcerative lesion (Yes/No)**	2.15	1.10–4.20	0.024
**Alcohol use (Yes vs. No)**	1.42	0.76–2.65	0.270

Legend: *p*-value = probability value.

**Table 3:** Multivariate logistic regression analysis of factors associated with in-hospital mortality in NVUGIB (n = 364).

Variable	Odds Ratio (OR)	95% Confidence Interval	*p*-Value
**Age (years)**	1.04	1.01–1.07	0.011
**Hemoglobin (g/dL)**	0.82	0.69–0.97	0.017
**Alcohol use (Yes vs. No)**	2.15	1.18–3.91	0.013
**Anticoagulant use (Yes vs. No)**	2.45	1.21–4.98	0.012
**Surgical intervention (Yes)**	2.92	1.32–6.48	0.008

Legend: *p*-value = probability value.

**Table 4:** Statistical comparison of clinical and therapeutic variables in relation to patient outcomes in non-variceal upper gastrointestinal bleeding.

Comparison	Test Type	Value	*p*-Value	Statistical Significance
**Mean age: deceased vs. survivors**	Student’s *t*-test	t = 3.51	0.0005	significant
**Mortality: alcohol users vs. non-users**	Chi-square	χ^2^ = 2.70	0.1001	not significant
**Type of antisecretory therapy**	Chi-square	χ^2^ = 19.36	0.0001	significant
**Type of endoscopic treatment**	Chi-square	χ^2^ = 4.79	0.0911	borderline
**Surgery timing intervals**	Chi-square	χ^2^ = 9.19	0.0565	borderline
**Types of surgical interventions**	Chi-square	χ^2^ = 33.71	<0.0001	significant

Legend: *p*-value = probability value, Student’s *t*-test = a statistical test used to compare the means of two groups, Chi-square = a statistical test used to determine whether there is a significant association between categorical variables, χ^2^—Chi-square statistic, a measure used to evaluate the association between categorical variables. Statistical significance—interpretation of *p*-value results: significant (*p* < 0.05), not significant (*p* ≥ 0.05), borderline (*p* close to 0.05).

## Data Availability

The data presented in this study are available on request from the corresponding author.
